# Features of Computer-Based Decision Aids: Systematic Review, Thematic Synthesis, and Meta-Analyses

**DOI:** 10.2196/jmir.4982

**Published:** 2016-01-26

**Authors:** Ania Syrowatka, Dörthe Krömker, Ari N Meguerditchian, Robyn Tamblyn

**Affiliations:** ^1^ Clinical and Health Informatics Research Group McGill University Montreal, QC Canada; ^2^ Department of Epidemiology, Biostatistics and Occupational Health McGill University Montreal, QC Canada; ^3^ McGill Centre for the Convergence of Health and Economics McGill University Montreal, QC Canada; ^4^ Department of Surgery McGill University Health Centre Montreal, QC Canada; ^5^ Department of Oncology McGill University Health Centre Montreal, QC Canada; ^6^ Department of Medicine McGill University Montreal, QC Canada

**Keywords:** computers, decision making, decision support systems, clinical, internet, medical informatics, patient participation, patient preference, patients

## Abstract

**Background:**

Patient information and education, such as decision aids, are gradually moving toward online, computer-based environments. Considerable research has been conducted to guide content and presentation of decision aids. However, given the relatively new shift to computer-based support, little attention has been given to how multimedia and interactivity can improve upon paper-based decision aids.

**Objective:**

The first objective of this review was to summarize published literature into a proposed classification of features that have been integrated into computer-based decision aids. Building on this classification, the second objective was to assess whether integration of specific features was associated with higher-quality decision making.

**Methods:**

Relevant studies were located by searching MEDLINE, Embase, CINAHL, and CENTRAL databases. The review identified studies that evaluated computer-based decision aids for adults faced with preference-sensitive medical decisions and reported quality of decision-making outcomes. A thematic synthesis was conducted to develop the classification of features. Subsequently, meta-analyses were conducted based on standardized mean differences (SMD) from randomized controlled trials (RCTs) that reported knowledge or decisional conflict. Further subgroup analyses compared pooled SMDs for decision aids that incorporated a specific feature to other computer-based decision aids that did not incorporate the feature, to assess whether specific features improved quality of decision making.

**Results:**

Of 3541 unique publications, 58 studies met the target criteria and were included in the thematic synthesis. The synthesis identified six features: content control, tailoring, patient narratives, explicit values clarification, feedback, and social support. A subset of 26 RCTs from the thematic synthesis was used to conduct the meta-analyses. As expected, computer-based decision aids performed better than usual care or alternative aids; however, some features performed better than others. Integration of content control improved quality of decision making (SMD 0.59 vs 0.23 for knowledge; SMD 0.39 vs 0.29 for decisional conflict). In contrast, tailoring reduced quality of decision making (SMD 0.40 vs 0.71 for knowledge; SMD 0.25 vs 0.52 for decisional conflict). Similarly, patient narratives also reduced quality of decision making (SMD 0.43 vs 0.65 for knowledge; SMD 0.17 vs 0.46 for decisional conflict). Results were varied for different types of explicit values clarification, feedback, and social support.

**Conclusions:**

Integration of media rich or interactive features into computer-based decision aids can improve quality of preference-sensitive decision making. However, this is an emerging field with limited evidence to guide use. The systematic review and thematic synthesis identified features that have been integrated into available computer-based decision aids, in an effort to facilitate reporting of these features and to promote integration of such features into decision aids. The meta-analyses and associated subgroup analyses provide preliminary evidence to support integration of specific features into future decision aids. Further research can focus on clarifying independent contributions of specific features through experimental designs and refining the designs of features to improve effectiveness.

##  Introduction

Over the past decade, health care has shifted from paper-based practice to electronic health records [1]. Patient information and education, such as decision aids, are also gradually moving toward online, computer-based environments [2]. Decision aids are support tools intended to help patients engage in high-quality decision making for preference-sensitive decisions [3]. Preference-sensitive decisions are cases where there is more than one clinically appropriate option (eg, choosing between mastectomy and lumpectomy for treatment of breast cancer [4]), and as a result, depend on patients’ values and preferences [3,5]. High-quality decision making occurs when well-informed patients consider evidence in the context of personal values and preferences to make a health decision. Decision aids have been shown to be effective for improving quality of decision making [3].

Considerable research has been conducted to guide content and presentation of decision aids [2,6-18]. In addition, the International Patient Decision Aid Standards Collaboration has established quality criteria for development and assessment of decision aids: (1) providing information in sufficient detail, (2) presenting probabilities in an unbiased manner, (3) including methods to clarify values and preferences, (4) providing structured guidance for deliberation and communication, (5) presenting information in a balanced manner, (6) using a systematic development process, (7) using up-to-date evidence, (8) disclosing conflicts of interest, (9) using plain language, and (10) ensuring that the decision is informed and values-based [6]. Additional quality criteria are recommended if the decision aid is delivered on the Internet or uses narratives. Further research has been conducted to expand upon these criteria. For example, risk communication literature builds on these standards by guiding how risk information (eg, probabilities) should be presented to facilitate high-quality decision making [19]. Similarly, research has been expanding around patient narratives [20,21] and explicit methods to clarify values and preferences [22]. However, given the relatively new shift to computer-based support, little attention has been given to how multimedia and interactivity can improve upon paper-based decision aids [2].

Theory suggests that integration of media rich or interactive features into computer-based decision aids can have a positive impact on quality of decision making by engaging patients in decision making beyond traditional static approaches [2]. Therefore, this review focuses on features that can be implemented only on electronic platforms and are not feasible in paper-based decision aids. The first objective of this review was to summarize published literature into a proposed classification of features that have been integrated into computer-based decision aids. Building on this classification, the second objective was to assess whether integration of specific features was associated with higher-quality decision making.

## Methods

### Study Selection

#### Search Strategy

Four databases (MEDLINE, Embase, CINAHL, and CENTRAL) were searched for all relevant studies published from 1946-2013. Three main concepts of decision support, the patient, and computer were mapped to the most relevant controlled vocabulary using Medical Subject Headings (MeSH), and free-text terms were added where necessary. Full search strategies are outlined in Multimedia Appendix 1.

#### Inclusion and Exclusion Criteria

The review identified studies that evaluated computer-based decision aids for adults faced with a preference-sensitive medical decision (ie, treatment, risk management, screening, or prevention) and that reported at least one quality of decision-making outcome (ie, knowledge, decisional conflict [23], decisional certainty, satisfaction with decision making, or decisional self-efficacy). All comparative study designs were considered; eligible controls included alternative aids and no decision support (eg, usual care or pre-post assessments). Studies were excluded if the article did not report original research, was not published in the English language, or if the decision aid was intended for proxy decision making.

#### Screening and Data Abstraction

Screening of articles was completed in two stages. Articles were first screened for relevance based on the information provided in the title and abstract and were then evaluated for inclusion based on the full text. Two reviewers independently screened articles at each stage (AS and DK). Disagreements were resolved by discussion and consensus between the 2 reviewers. Overall kappa score was calculated to assess interrater reliability [24].

One reviewer completed data abstraction (AS), which focused on citation information, study design, decision context, interventions, controls (eg, usual care or alternative aids), components being tested, and quality of decision-making outcomes. If an article included in the review cited a development paper or webpage, then information from these sources was used to supplement data abstracted from the article.

### Thematic Synthesis

All studies identified for the systematic review were included in the thematic analysis. Data abstracted from the articles was used to create a proposed classification of features that have been integrated into computer-based decision aids to date. The classification was developed based on logical groupings and informed by themes from decision aid literature [6,7].

Selection of groupings was completed by 1 reviewer (AS), guided by steps outlined by Thomas & Harden for conducting thematic analysis: (1) line-by-line coding of articles to record components, (2) development of descriptive themes, and (3) creation of analytical themes [25]. Components were grouped into types (ie, descriptive themes) if the function of the components was similar; for example, components that improved navigation were grouped together. Similarly, types of components were judged to be similar and grouped into features (ie, analytical themes) if they served a similar purpose; for example, components that improved access to information were grouped together within the same feature. The proposed features classification went through an iterative process of review by co-authors and colleagues and was revised accordingly.

### Meta-Analyses

Only randomized controlled trials (RCTs) that reported knowledge or decisional conflict were included in the quantitative synthesis. Decisional certainty, satisfaction with decision making, and decisional self-efficacy were not included due to the lower number of studies that reported these outcomes. The standardized mean difference (SMD; ie, Cohen’s *d* effect size) was calculated for each study [26]. For studies that reported multiple comparisons, only the comparison that tested the highest number of features was included in the analysis. If multiple measures were taken over time, only the first measure of knowledge or decisional conflict was included in the analysis. If studies reported more than one type of knowledge or more than one subscale of the decisional conflict scale (without reporting the total), then an average of the SMDs was calculated. For articles that did not provide sufficient information, study authors were contacted for additional information required to calculate the SMD.

The overall effect of computer-based decision aids was estimated by pooling the SMD of each study using Review Manager (version 5.3). Studies were pooled using inverse variance weighting and random effects models with 95% confidence intervals. Heterogeneity of pooled SMDs was assessed based on I^2^ statistics [27].

### Tests for Subgroup Differences

Subgroup analyses were conducted to test whether specific features (or types of components) could explain some of the heterogeneity in the overall effect. Subgroup analyses compared pooled SMDs for decision aids that incorporated a specific feature to other computer-based decision aids that did not incorporate the feature to assess whether specific features were associated with improvements in quality of decision making. The Review Manager test for subgroup differences was used to assess statistical significance.

## Results

### Study Selection

**Figure 1:**
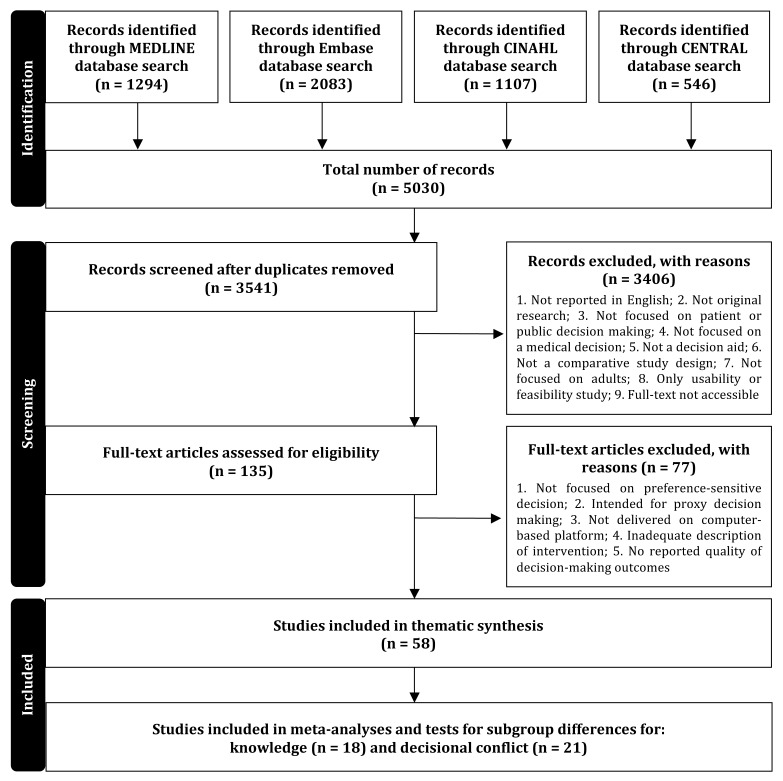
Modified PRISMA study selection flowchart.

The search identified 3541 eligible articles. The title and abstract screen retained 135 articles. Full text screening identified 58 studies that met the target criteria and were included in the thematic synthesis. The overall kappa score for screening was 0.60, reflecting moderate interrater agreement [28]. A subset of 26 RCTs from the thematic synthesis was used to conduct the meta-analyses with 18 of the articles reporting knowledge and 21 of the articles reporting decisional conflict. A modified Preferred Reporting Items for Systematic Reviews and Meta-Analyses (PRISMA) flowchart is presented in Figure 1 [29], and studies included in the meta-analyses are indicated in Multimedia Appendix 2.

Data abstracted from articles are presented in Multimedia Appendix 2, including citation information, study design, decision context, interventions, controls, components being tested, and SMDs for RCTs reporting knowledge or decisional conflict [30-101]. The studies were published between 1996 and 2013 and came primarily from the United States (37/58, 64%), Canada (7/58, 12%), and the United Kingdom (7/58, 12%). The majority (33/58, 57%) of decision aids were developed for treatment decisions. The remaining decision aids were created for risk management (6/58, 10%), screening (15/58, 26%), and prevention decisions (5/58, 9%).

Studies assessed quality of decision making by measuring knowledge (36/58, 62%), decisional conflict (30/58, 52%), decisional certainty (21/58, 36%), satisfaction with decision making (16/58, 28%), and decisional self-efficacy (7/58, 12%). Studies compared computer-based decision aid performance to usual care (18/58, 31%), alternative aids (29/58, 50%), or based on pre-assessments (14/58, 24%).

### Thematic Synthesis

The thematic analysis identified six main features that have been integrated into computer-based decision aids: content control, tailoring, patient narratives, explicit values clarification, feedback, and social support. A proposed classification for these features and types of components is presented in Table 1. References to decision aids that implemented these features, as well as mode of presentation for each decision aid (eg, Web-based, videobooklet, CD-ROM) are provided in Multimedia Appendix 2, under columns titled “Features being tested” and “Interventions,” respectively.

**Table 1:** Proposed classification of features that have been integrated into computer-based decision aids (58 studies).

Features	Types of components	Examples of components
Content control: Patient has control over access to information	Navigation	Menu bar, search function, television-like interface, touchscreen, help menu
	Clarity of information	Glossary, information summaries, supplementary risk diagrams, metaphors, narration
	Optional information	“Learn more” sections for detailed information about topics of interest
	Access to external resources	Reference lists, links to summaries of recent studies or clinical practice guidelines, developer contact information
Tailoring: Patient receives personalized information	Demographics	Patient age, gender, race/ethnicity, family history, health literacy
	Clinical condition	Specific diagnosis, stage of disease, comorbidities, current symptoms, current medications, past treatment experience, eligibility for interventions
	Values, preferences, and beliefs	Language, preferred role in decision making, stage of decision making, preference for colloquial vs technical terms, beliefs around efficacy of screening or treatment
	Knowledge deficits	Focus on information that is unclear or incorrect based on knowledge pre-tests
Patient narratives: Patient reflects on experiences of others	Patient stories (focus on personal experiences)	Video of patient sharing personal experience
	Behavior modeling (focus on process of deliberation)	Video of patient weighing options, video vignettes of common concerns around decision making
Explicit values clarification: Patient examines personal values and preferences	Decision points	Strategically placed questions to determine whether patient is prepared to move forward to next section of decision aid
	Notebook	Memory aid used to store issues of concern, “bookmarks” for important sections
	Weighting exercises	Simple yes/no questions, feeling thermometer, balance scale, selecting initial treatment decision
	Trade-off exercises	Simple rank order exercises, adaptive conjoint analysis-based tools
	Social matching	“Soap opera” episodes with questions to determine which character embodies patient’s values and preferences
	Personal reflection	Patient considers perspectives of others affected by the decision (eg, partners, family members, or others)
Feedback: Patient receives important information around decision making based on interactions with aid	Decision aid progress	Program tracks information that has been covered, and suggests important information that has not been accessed
	Knowledge	Self-evaluations provide feedback on comprehension of evidence presented
	Summary of preferences	Bar graphs depicting relative importance of personal values and preferences
	Optimal choice	Patient values and preferences are incorporated into an algorithm to determine the most suitable option
	Decisional consistency	Alerts patient if initial treatment decision is not consistent with optimal choice
	Summary of decision aid activity (usually printed)	Plan of action based on initial treatment decision, personal risk summaries
Social support: Patient encouraged to involve others in decision-making	Community support	Celebrity endorsement, video of patient celebration after completing treatment, links to support groups
	Integration of family	Modules specific to others affected by the decision, information on how to communicate with partner
	Facilitation of shared decision making	Video of physician describing options and outcomes, video of physician encouraging patient to adhere to chosen option, video coaching to overcome physician communication barriers, recommended questions for physician consultations, copy of decision aid summary placed in patient chart, physician-specific modules

The majority of studies included in the thematic analysis provided content control (42/58, 72%). Two-thirds tailored information to the patient (38/58, 66%), and almost half incorporated patient narratives (28/58, 48%). Over half of the studies provided explicit values clarification (31/58, 53%), feedback (36/58, 62%), or social support (32/58, 55%). One third of the studies incorporated five (13/58, 22%) or all six (10/58, 17%) of these features.

### Meta-Analyses

**Figure 2:**
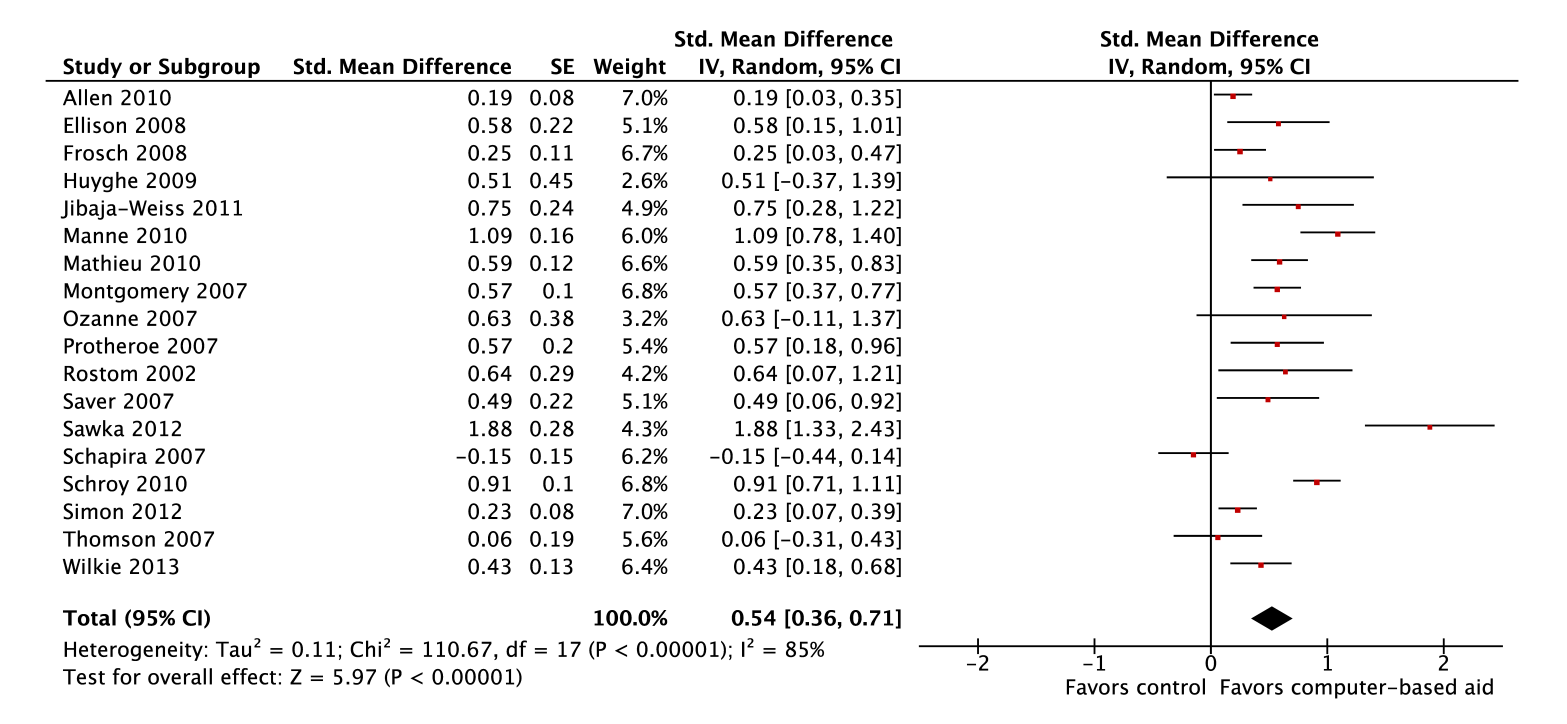
Forest plot of SMDs for improvements in knowledge (18 studies).

Eighteen studies were included in the meta-analysis to assess whether or not use of computer-based decision aids improved knowledge. The studies included were published between 2001 and 2013. Most computer-based decision aids performed significantly better than usual care or alternative aid controls (14/18, 78%); the performance of the remaining decision aids was not significantly different from controls. Overall, computer-based decision aids were associated with significant improvements in knowledge with a pooled SMD of 0.54 (95% CI 0.36-0.71; *P*<.001). A forest plot is presented in Figure 2.

**Figure 3:**
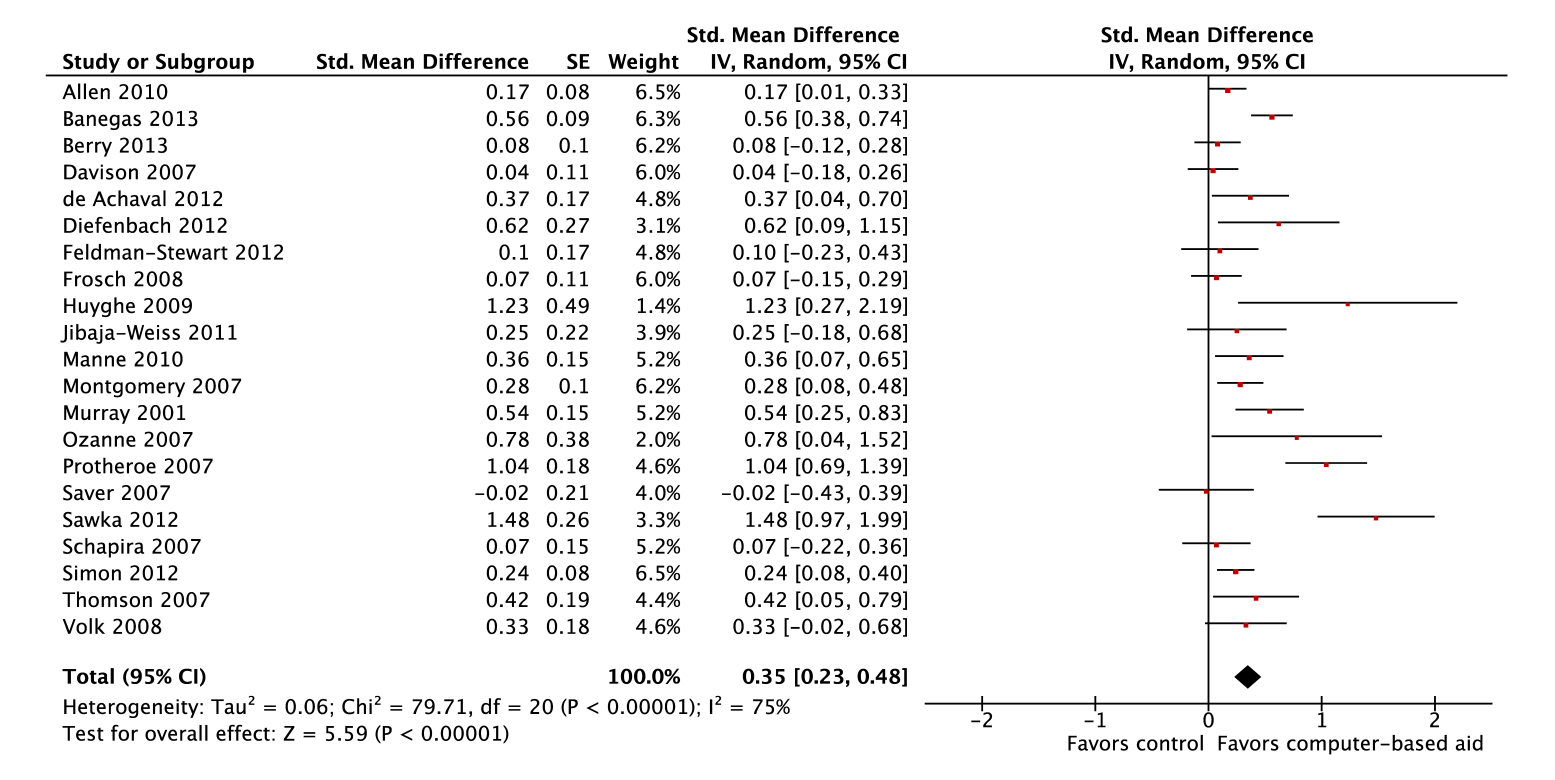
Forest plot of SMDs for improvements in decisional conflict (21 studies).

We included 21 studies in the meta-analysis to assess whether or not use of computer-based decision aids improved decisional conflict. The studies included were published between 2002 and 2013. Most computer-based decision aids performed significantly better than usual care or alternative aid controls (13/21, 62%); the performance of the remaining decision aids was not significantly different from controls. Overall, computer-based decision aids were associated with significant improvements in decisional conflict with a pooled SMD of 0.35 (95% CI 0.23-0.48; *P*<.001). A forest plot is presented in Figure 3.

Although computer-based decision aids performed significantly better than usual care or alternative aids, there was a high level of heterogeneity in study-level SMDs. The I^2^ statistics were 84% and 75% for knowledge and decisional conflict, respectively.

### Tests for Subgroup Differences

The six features and associated types identified through the thematic analysis were used to inform subgroup analyses. The results are presented in Tables 2 (for knowledge) and 3 (for decisional conflict), which present and compare pooled SMDs for decision aids that include a specific feature (or type) to other computer-based decision aids that do not include the feature. The number of studies included in each subgroup and *P* values associated with the Review Manager test for subgroup differences are also reported.

Overall, integration of content control was positively associated with quality of decision making, although the association was only significant for knowledge (*P*=.008). However, specific types had differing effects. Knowledge and decisional conflict were negatively affected by offering navigation (*P*=.56, *P*=.08, respectively), whereas knowledge and decisional conflict both improved by providing clarity around evidence (*P*=.03, *P*=.07, respectively), optional in-depth information (*P*=.05, *P*=.42, respectively), or access to external resources (*P*=.65, *P*=.15, respectively).

Conversely, tailoring was negatively associated with knowledge and decisional conflict (*P*=.08, *P*=.07, respectively). This association was consistent across all types; both knowledge and decisional conflict were negatively affected by tailoring based on patient demographics (*P*=.07, *P*=.31, respectively), clinical condition (*P*=.06, *P*=.14, respectively), or values, preferences, and beliefs (*P*=.14, *P*=.02, respectively).

Similarly, patient narratives reduced quality of decision making; however, the association was significant only for decisional conflict (*P*=.005). Both knowledge and decisional conflict were negatively affected by presenting patient stories (*P*=.54, *P*=.11, respectively), or behavior modeling (*P*=.32, *P*=.01, respectively).

Explicit values clarification reduced knowledge (*P*=.42) but did not affect decisional conflict. However, effects varied for different types. Both knowledge and decisional conflict improved by integrating notebooks (*P*=.68, *P*=.56, respectively), or trade-off exercises (*P*=.84, *P*=.60, respectively). Knowledge was reduced by providing weighting exercises (*P*=.18) or social matching (*P*=.47), with no effect on decisional conflict. Likewise, personal reflection reduced knowledge (*P*=.47).

Overall, providing feedback was negatively associated with knowledge and decisional conflict (*P*=.40, *P*=.63, respectively). Again, effects varied by type. Reporting progress through the decision aid improved decisional conflict (*P*=.32). Likewise, both knowledge and decisional conflict improved by providing knowledge feedback (*P*=.80, *P*=.07, respectively). Providing a summary of preferences did not affect decisional conflict. Optimal choice feedback reduced knowledge (*P*=.44), but improved decisional conflict (*P*=.54). Both knowledge and decisional conflict were negatively affected by providing feedback around decisional consistency (*P*<.001, *P*=.31, respectively), or providing a summary of decision aid activity (*P*=.35, *P*=.62, respectively).

**Table 2:** Number of studies and pooled SMDs for improvements in knowledge comparing decision aids including each feature to decision aids not including the feature (18 studies).

Feature and types of components		Studies, n	Feature included, pooled SMD (95% CI)	Studies, n	Reference (no feature), pooled SMD (95% CI)	*P* value^a^
Overall: Any feature		18	0.54 (0.36-0.71)	0	—	—
**Content control**		15	0.59 (0.39-0.79)	3	0.23 (0.05-0.41)	.008
	Navigation	7	0.47 (0.19-0.76)	11	0.59 (0.34-0.83)	.56
	Clarity of information	13	0.65 (0.44-0.87)	5	0.24 (-0.05-0.54)	.03
	Optional information	7	0.76 (0.42-1.09)	11	0.38 (0.21-0.54)	.05
	Access to external resources	6	0.63 (0.15-1.10)	12	0.51 (0.32-0.70)	.65
**Tailoring**		10	0.40 (0.18-0.62)	8	0.71 (0.44-0.99)	.08
	Demographics	9	0.38 (0.15-0.62)	9	0.71 (0.45-0.96)	.07
	Clinical condition	8	0.36 (0.11-0.61)	10	0.69 (0.46-0.93)	.06
	Values, preferences, and beliefs	3	0.31 (0.00-0.62)	15	0.59 (0.38-0.79)	.14
	Knowledge deficits	0	—	18	0.54 (0.36-0.71)	—
**Patient narratives**		8	0.43 (0.19-0.68)	10	0.65 (0.37-0.93)	.26
	Patient stories	7	0.47 (0.20-0.75)	11	0.59 (0.34-0.83)	.54
	Behavior modeling	3	0.39 (0.11-0.67)	15	0.57 (0.36-0.78)	.32
**Explicit values clarification**		11	0.48 (0.30-0.65)	7	0.67 (0.23-1.12)	.42
	Decision points	0	—	18	0.54 (0.36-0.71)	—
	Notebook	3	0.59 (0.35-0.84)	15	0.53 (0.33-0.73)	.68
	Weighting exercises	8	0.41 (0.24-0.58)	10	0.65 (0.34-0.95)	.18
	Trade-off exercises	3	0.58 (0.12-1.04)	15	0.53 (0.33-0.72)	.84
	Social matching	1	0.43 (0.18-0.68)	17	0.55 (0.36-0.73)	.47
	Personal reflection	1	0.43 (0.18-0.68)	17	0.55 (0.36-0.73)	.47
**Feedback**		8	0.46 (0.27-0.64)	10	0.60 (0.31-0.89)	.40
	Decision aid progress	0	—	18	0.54 (0.36-0.71)	—
	Knowledge	2	0.60 (0.12-1.08)	16	0.53 (0.35-0.72)	.80
	Summary of preferences	0	—	18	0.54 (0.36-0.71)	—
	Optimal choice	3	0.42 (0.11-0.73)	15	0.57 (0.36-0.78)	.44
	Decisional consistency	2	0.17 (0.03-0.31)	16	0.60 (0.40-0.79)	<.001
	Summary of decision aid activity	6	0.44 (0.23-0.65)	12	0.60 (0.34-0.86)	.35
**Social support**		10	0.58 (0.32-0.84)	8	0.50 (0.23-0.76)	.67
	Community support	4	0.91 (0.34-1.48)	14	0.45 (0.27-0.63)	.14
	Integration of family	3	0.50 (0.29-0.72)	15	0.54 (0.34-0.74)	.82
	Facilitation of shared decision making	6	0.44 (0.13-0.75)	12	0.59 (0.36-0.82)	.45

^a^Review Manager test for subgroup differences.

**Table 3:** Number of studies and pooled SMDs for improvements in decisional conflict comparing decision aids including each feature to decision aids not including the feature (21 studies).

Feature and types of components		Studies, n	Feature included, pooled SMD (95% CI)	Studies, n	Reference (no feature), pooled SMD (95% CI)	*P* value^a^
Overall: Any feature		21	0.35 (0.23-0.48)	0	—	—
**Content control**		14	0.39 (0.23-0.56)	7	0.29 (0.08-0.49)	.42
	Navigation	8	0.22 (0.10-0.34)	13	0.42 (0.23-0.60)	.08
	Clarity of information	12	0.46 (0.28-0.65)	9	0.23 (0.07-0.40)	.07
	Optional information	6	0.44 (0.20-0.68)	15	0.32 (0.17-0.47)	.42
	Access to external resources	5	0.72 (0.12-1.33)	16	0.28 (0.18-0.37)	.15
**Tailoring**		12	0.25 (0.13-0.37)	9	0.52 (0.26-0.79)	.07
	Demographics	10	0.29 (0.16-0.42)	11	0.43 (0.20-0.65)	.31
	Clinical condition	10	0.26 (0.12-0.40)	11	0.46 (0.23-0.68)	.14
	Values, preferences, and beliefs	7	0.18 (0.07-0.30)	14	0.44 (0.27-0.61)	.02
	Knowledge deficits	0	—	21	0.35 (0.23-0.48)	—
**Patient narratives**		8	0.17 (0.08-0.26)	13	0.46 (0.28-0.65)	.005
	Patient stories	5	0.20 (0.03-0.38)	16	0.39 (0.24-0.54)	.11
	Behavior modeling	4	0.16 (0.05-0.27)	17	0.41 (0.25-0.56)	.01
**Explicit values clarification**		13	0.36 (0.20-0.51)	8	0.36 (0.14-0.58)	.97
	Decision points	0	—	21	0.35 (0.23-0.48)	—
	Notebook	4	0.48 (-0.02 to 0.98)	17	0.32 (0.20-0.44)	.56
	Weighting exercises	9	0.35 (0.16-0.53)	12	0.36 (0.19-0.54)	.89
	Trade-off exercises	3	0.48 (-0.08 to 1.04)	18	0.33 (0.20-0.45)	.60
	Social matching	1	0.33 (-0.02 to 0.68)	20	0.36 (0.23-0.49)	.89
	Personal reflection	0	—	21	0.35 (0.23-0.48)	—
**Feedback**		11	0.32 (0.16-0.49)	10	0.39 (0.19-0.58)	.63
	Decision aid progress	1	0.62 (0.09-1.15)	20	0.35 (0.22-0.47)	.32
	Knowledge	1	1.23 (0.27-2.19)	20	0.34 (0.22-0.46)	.07
	Summary of preferences	1	0.37 (0.04-0.70)	20	0.35 (0.23-0.48)	.93
	Optimal choice	4	0.45 (0.09-0.81)	17	0.33 (0.19-0.46)	.54
	Decisional consistency	2	0.24 (0.02-0.45)	19	0.37 (0.23-0.51)	.31
	Summary of decision aid activity	9	0.32 (0.15-0.50)	12	0.39 (0.20-0.57)	.62
**Social support**		11	0.38 (0.19-0.57)	10	0.34 (0.17-0.51)	.75
	Community support	4	0.50 (-0.08 to 1.07)	17	0.33 (0.21-0.45)	.58
	Integration of family	2	0.64 (-0.30 to 1.58)	19	0.35 (0.22-0.47)	.54
	Facilitation of shared decision making	8	0.29 (0.13-0.45)	13	0.38 (0.21-0.56)	.46

^a^Review Manager test for subgroup differences.

Social support improved knowledge (*P*=.67) but did not affect decisional conflict. Both knowledge and decisional conflict improved by providing community support (*P*=.14, *P*=.58, respectively). Integration of family support did not affect knowledge but improved decisional conflict (*P*=.54). Both knowledge and decisional conflict were negatively affected by facilitation of shared decision making (*P*=.45, *P*=.46, respectively).

## Discussion

### Principal Results

This review summarizes published literature into a proposed classification of features that have been integrated into computer-based decision aids. The thematic synthesis identified six main features of content control, tailoring, patient narratives, explicit values clarification, feedback, and social support. Building on this classification, meta-analyses with tests for subgroup differences were conducted to evaluate whether specific features improved quality of decision making. Overall, decision aids that integrated these features performed significantly better than usual care or alternative aids. The exploratory subgroup analyses rank-ordered the features. Overall, content control performed better than other features. Conversely, tailoring and patient narratives performed worse compared to other features. Results were varied for different types of explicit values clarification, feedback, and social support.

### Thematic Synthesis

The proposed features classification is the first of its kind for decision aids. It serves two purposes: to provide the first step towards improving reporting of features that are integrated into computer-based decision aids and to promote use of such features in future decision aids. Currently, reporting standards for interventions are specific about the overarching goal of replicability; however, they offer little guidance around how to reach this goal. For example, the Consolidated Standards of Reporting Trials (CONSORT) statement simply states that authors should report sufficient information to ensure replicability, including detail around how and when interventions were administered [102]. The CONSORT statement extension for nonpharmacologic interventions builds on the former by stating that authors should report different components of interventions and procedures for tailoring interventions [103]. As a result of such vague recommendations, published descriptions of interventions, including decision aids, often do not provide sufficient information to guide replication [104]. In addition, lack of consistent language in reporting means that similar interventions can be reported using different descriptions, which can complicate evaluations to identify features that make interventions successful. Consequently, there has been a call for better reporting guidance [104]. To address such limitations in the field of behavior change, Abraham & Michie developed a classification of active behavior change interventions [105]. Using the classification, a systematic review and meta-regression were conducted to evaluate behavior change interventions to promote healthy eating or physical activity [106]. The analysis highlighted “active ingredients” of successful interventions that may have otherwise been missed. Similarly, the classification proposed in this study can lead to better reporting of features that are integrated into computer-based decision aids, using consistent language. Improved reporting will build further evidence around the value of these features, which can guide integration of these features into future decision aids.

### Meta-Analyses

As expected, computer-based decision aids were associated with significant improvements in knowledge and decisional conflict compared to usual care or alternative aids [3]. The subgroup analyses served as exploratory assessments of specific features and provided insight into which features perform better than others.

### Tests for Subgroup Differences

#### Content Control

Overall, content control improved quality of decision making. All types of content control performed better than other features, with the exception of navigation. Content control is intended to provide patients with control over order, detail, and type of evidence presented [2]. The concept of content control is promising, considering that patients have different preferences for the amount of detail presented [107]. In addition, these preferences, often correlating with health literacy, can change over time, which highlights that a one-size-fits-all solution is not appropriate [107]. Ideally, content control should improve engagement, which has the potential to facilitate understanding and retention of evidence. In addition, providing the means to self-personalize content may promote autonomy and empower patients to take ownership over health care decisions.

Interestingly, navigation reduced quality of decision making compared to other features. Given that navigation is a foundational piece of computer-based interventions, this may represent a reporting bias. As a result of journal space limitations, navigation may have been underreported in exchange for reporting novel or impressive decision aid components. This relates back to the need for a classification to ensure that all features are reported; otherwise, important features may be overlooked as a result of biased evaluations.

#### Tailoring

Tailoring reduced quality of decision making, with all subgroups performing worse than other features. In general, tailoring is intended to translate evidence into patient-specific information to improve engagement. The effects of tailoring can be split into two categories: (1) effects on calculation of risk estimates, and (2) effects on presentation of information.

Tailoring can be used to frame evidence in terms of patient demographics or clinical condition to present only viable treatment options with more accurate estimates of associated risks and benefits. Ideally, this should provide a better understanding of personal situations and lead to high-quality decision making. However, evidence around the benefits of tailoring risk estimates is varied [13]. Tailoring may not be effective or may even be detrimental to decision making if personalized risk estimates are not considered appropriate by the patient [108]. Patients may distrust risk estimates if the estimates are not congruent with previous knowledge or if the risk calculations are not transparent or omit risk factors perceived as important [108]. Most of the tailoring incorporated in the decision aids included in this study was superficial and did not present risk information in the context of population risks. It is possible that the tailoring approaches were not developed appropriately and did not tailor sufficiently to be considered useful by the patients.

Tailoring can also be used to present evidence in terms of patient preferences or to address knowledge deficits, in an effort to facilitate understanding or to correct misinformation. However, this form of tailoring may limit the amount or type of evidence that is presented. For example, decision aids can be tailored to information-seeking style (ie, high or low levels of detail) [30] or information preferences (ie, focus on topics of interest) [31]. Such tailoring approaches may result in an incomplete or unbalanced presentation of evidence and unintentionally reduce quality of decision making. In fact, this form of tailoring could be considered the reverse of content control. Specifically, content control may promote patient autonomy by providing control over evidence reviewed, whereas tailoring employs a paternalistic model of presenting evidence, and as a result, may reduce autonomy.

#### Patient Narratives

Similarly, patient narratives reduced quality of decision making. Patient narratives are intended to provide insight into patient experiences and bring attention to important evidence to consider throughout the decision-making process. In addition, information presented through patient narratives is processed differently than written information and can improve understanding and retention of evidence [15,20,109]. However, narratives can unintentionally present biased or unbalanced information, which may result in lower-quality decision making [2,20,110]. To date, there is no consensus around the value of including patient narratives in decision aids [2,15].

In this study, both patient stories and behavior modeling scenarios reduced quality of decision making. Shaffer and Zikmund-Fisher have developed a taxonomy for patient narratives outlining dimensions that are expected to impact decision making: (1) purpose of the narrative, (2) content of the narrative, and (3) evaluative valence (ie, tone of the narrative) [20]. This taxonomy highlights different types of content and proposes underlying mechanisms on how content might affect decision making for outcome, experience, and process narratives. Given that most articles simply reported inclusion of patient narratives, it is unclear whether the dimensions of purpose, content, and tone can explain the reduction in quality of decision making. However, based on this taxonomy, if the content of patient stories was disproportionally focused on adverse events, then risks associated with selecting a particular option may have been overrepresented. If anecdotal information presented through patient stories contradicts statistical data presented in the decision aid, then these conflicting messages may have reduced quality of decision making. Behavior modeling narratives (similar to process narratives described in the taxonomy) are intended to increase deliberation, by guiding the patient to consider or disregard specific factors related to decision making. However, if a narrative places more emphasis on factors not considered to be important or relevant to the patient, this could impede the decision-making process. This may even lead to lower-quality decision making by encouraging patients to focus on factors that are not in line with personal values or preferences. Further research can help determine the value of specific types of patient narratives, and what type of content and presentation facilitate higher-quality decision making.

The negative effects of tailoring and patient narratives on quality of decision making in decision aids were unexpected, considering the positive impact of tailoring and patient narratives when employed in behavior change interventions [111-113]. A potential reason for this may lie in the fundamental differences in purpose between decision aids and behavior change interventions. Specifically, behavior change interventions are intended to persuade the user to take up a specific health behavior, which is considered to be the best option [114]. However, this is not the purpose of decision aids, which are developed for preference-sensitive decisions where there is more than one clinically appropriate option [3]. The overarching goal of decision aids is to provide complete, balanced, and unbiased information to facilitate high-quality decision making. Tailoring and patient narratives, as implemented in the decision aids included in this systematic review, may have unintentionally presented superficial, unbalanced, or biased information, which may have reduced the quality of decision making. Further research needs to be conducted to refine the content and presentation of these two features to improve their effectiveness in decision aids.

#### Explicit Values Clarification

Specific types of explicit values clarification had a positive effect on quality of decision making. Explicit values clarification methods are intended to guide patients through specific tasks to identify personal values and preferences [14]. Few have been evaluated, and findings are mixed [14]. In this study, providing a “notebook” to record topics that were unclear or of concern was associated with higher-quality decision making. This device allowed patients to highlight areas where they needed more information, or where they needed to reflect more deeply on values and preferences. As a result, patients were more knowledgeable and had lower decisional conflict. Likewise, trade-off exercises (eg, rank-ordering all outcomes from most to least important) also improved quality of decision making. Such exercises provide a realistic approach to decision making, where the patient must consider and trade-off between risks and benefits of two or more options. In contrast, weighting exercises (eg, ranking each outcome on a scale of 1-10) can be inadequate if the patient ranks everything as equally important. In essence, trade-off exercises may help to better clarify issues around decision making.

#### Feedback

Specific types of feedback were also associated with improvements in quality of decision making. Feedback is intended to provide the patient with important information around decision making based on interactions with the decision aid. Progress through the decision aid and knowledge feedback both improved quality of decision making. Both are intended to ensure that the patient is well informed by confirming that all necessary information is reviewed by the patient and to correct misinformation, respectively. Summary of preferences, optimal choice, and decisional consistency are types of feedback that are specific to explicit values clarification methods. Summary of preferences provides feedback around how patients personally value risks and benefits integral to decision making. Optimal choice builds on summary of preferences, by suggesting which option is best based on patients’ values and preferences, which had a positive effect on decisional conflict. Similarly, research has shown that providing implications of stated values (ie, optimal choice) may have a positive effect on decision making [22]. In contrast, decisional consistency reduced quality of decision making. This form of feedback elicits patients’ initial decisions and reports whether or not the initial decisions are consistent with patients’ values and preferences. This may lead to increased decisional conflict when the decision aid reports a lack of decisional consistency. This gives the impression that the initial decision was wrong and may elicit negative emotional responses from the patient.

#### Social Support

Specific types of social support improved quality of decision making. This feature is intended to reinforce that the patient is not alone in their experiences or decision making. Social support is a recurring theme throughout patient needs assessments for medical care [115-117], and patient decision aids specifically [118]. However, little guidance has been offered around what type of social supports can be integrated into decision aids and how. This review identified three types: community (ie, support from others faced with the same decision), family (ie, support from others affected by the specific decision), and clinician support (ie, facilitation of shared decision making). Community support improved knowledge and decisional conflict. Patients who had access to this type of support were likely able to have knowledge questions answered, as well as discuss how personal values and preferences fit into decision making. Integration of family support had positive effects on decisional conflict. Patients who had access to this type of support were likely able to better engage their partners and family members in the decision-making process and discuss values and preferences. Social support, specifically community and family support, seems to be promising but was limited by sample size in this study.

### Principles for Decision Aid Development and Future Directions

Based on the study findings, content control should be integrated into decision aids to allow patients to select the order, level of detail, and type of information presented. This approach allows the patient to directly access topics of interest, view alternative presentations of information for clarity, and access optional information or external resources. However, to ensure balanced representation of all options, it is important to integrate safeguards to ensure that the patient reviews all necessary evidence (ie, not “optional” information) prior to making a final decision.

Tailoring, as currently developed and presented, should be used with caution, as it may reduce quality of decision making. Ineffective tailoring may have resulted from superficial or non-transparent tailoring, which patients did not believe reflected their true risk. Allowing patients to “self-tailor” through content control may be a viable option until effective strategies for tailoring information are established.

Patient narratives should also be used with caution, as they may reduce quality of decision making. Patient narratives may unintentionally present unbalanced or biased information, which may undermine statistical data presented in the decision aid or encourage patients to focus on factors that are not in line with personal values or preferences. Further research should focus on identifying types of narrative content and presentation that facilitate quality decision making.

Further research is also needed in the areas of feedback, explicit values clarification, and social support to guide future integration. There was substantial heterogeneity in effects between types of components within each of these features, which may reflect artificial grouping of components. In addition, small sample sizes limited appropriate assessments, with many components having been tested only in one decision aid, which limited guidance for integration of these features into decision aids.

### Limitations

Studies included in the meta-analyses had a high level of heterogeneity with regard to patient populations, decision context, characteristics of the interventions, and components being tested, as well as choice of usual care or alternative aid controls. Studies were selected for inclusion based on testing a computer-based decision aid intervention, evaluating quality of decision making by measuring either knowledge or decisional conflict, and using an RCT design. For each subgroup analysis, decision aids that incorporated a specific feature (or type of component) were compared to decision aids that did not incorporate the feature. Effectively, this approach compared groupings of studies that tested various complex decision aids against very different control groups. Therefore, results from this study should be interpreted as “hypothesis-generating” and should be considered preliminary evidence to guide future work in this area.

Small numbers of studies incorporated certain types of components, which reduced the power to detect significant subgroup differences but also increased the probability of false positives. Nevertheless, subgroup analyses were conducted for all features and types of components for completion, and the number of studies in each subgroup was considered when interpreting the results.

Similarly, the study did not adjust for numerous comparisons generated through the subgroup analyses, which also increased the probability of false positives. However, given the exploratory nature of the study, such adjustments may not be necessary, since findings will require further research to establish independent contributions of each feature [27].

Given that the majority of decision aids incorporated multiple features, conducting subgroup analyses limited the capacity to disentangle the effects of specific features or to assess whether specific bundles of features were more effective for improving quality of decision making. Ideally, conducting a meta-regression, similar to the analysis described by Michie et al, would address these shortcomings [106]. However, a meta-regression was not considered appropriate for this study. The regression coefficients would have been unstable given the low number of studies (18 for knowledge and 21 for decisional conflict), and it would have been impossible to control for the substantial heterogeneity of decision aids included in the analysis. Therefore, straightforward exploratory subgroup analyses were selected as a viable alternative.

### Conclusions

Integration of media rich or interactive features into computer-based decision aids can improve quality of preference-sensitive decision making beyond traditional static approaches. However, this is an emerging field with limited evidence to guide implementation. The systematic review and thematic synthesis identified features used in available computer-based decision aids, in an effort to facilitate reporting of these features and to promote integration of such features into decision aids. The meta-analyses and associated subgroup analyses provide preliminary evidence to support integration of specific features into future decision aids. Further research can focus on clarifying independent contributions of specific features through experimental designs and refining the designs of features to improve effectiveness.

## Appendix

Multimedia Appendix 1 Systematic review search strategies for MEDLINE, Embase, CINAHL, and CENTRAL.

Multimedia Appendix 2 Information about studies included in the systematic review.

## Abbreviations

CONSORT: Consolidated Standards of Reporting Trials
MeSH: Medical Subject Headings
PRISMA: Preferred Reporting Items for Systematic Reviews and Meta-Analyses
RCT: randomized controlled trial
SMD: standardized mean difference
