# Supplementary material for: Features of Computer-Based Decision Aids: Systematic Review, Thematic Synthesis, and Meta-Analyses
Source: J Med Internet Res. 2016 Jan 26;18(1):e20. doi: 10.2196/jmir.4982 (PMC4748141; doi:10.2196/jmir.4982)
Supplement: Multimedia Appendix 1 [file jmir_v18i1e20_app1.pdf]

Multimedia Appendix 1: Systematic review search strategies for MEDLINE, Embase, CINAHL and CENTRAL

MEDLINE

The search strategy below was created and refined in MEDLINE with guidance from the McGill Health Sciences Library to identify studies for screening:

Database: Ovid MEDLINE(R), Ovid MEDLINE(R) In-Process & Other Non-Indexed Citations, Ovid MEDLINE(R) Daily and Ovid OLDMEDLINE(R) <1946 to Present>
Search Strategy:
----
1 decision support techniques/ (12180)
2 decision support systems, clinical/ (5126)
3 decision making/ (68890)
4 choice behavior/ (22189)
5 health communications/ (494)
6 audiovisual aids/ (6233)
7 decision support*.tw. (7830)
8 decision tool*.tw. (374)
9 1 or 2 or 3 or 4 or 5 or 6 or 7 or 8 (115018)
10 patient education as topic/ (71125)
11 exp patients/ (66854)
12 consumer participation/ (14427)
13 patient participation/ (18078)
14 patient satisfaction/ (59319)
15 patient preference/ (2687)
16 10 or 11 or 12 or 13 or 14 or 15 (219310)
17 9 and 16 (11503)
18 decision aid*.tw. (1406)
19 shared decision making.tw. (2208)
20 patient decision*.tw. (887)
21 17 or 18 or 19 or 20 (14228)
22 user-computer interface/ (30657)
23 medical informatics/ (8290)
24 internet/ (52671)
25 computers/ (48912)
26 computers, handheld/ (2339)
27 interactive.tw. (31942)
28 internet.tw. (29053)
29 web based.tw. (15331)
30 computer*.tw. (231959)
31 22 or 23 or 24 or 25 or 26 or 27 or 28 or 29 or 30 (372622)
32 21 and 31 (1339)
33 limit 32 to English language (1294)

The first block of MeSH and free text terms (lines 1 to 8) locates studies that offer decision support. Line 9 combines the MeSH and free text terms for the *decision support* concept with the Boolean operator "OR".

The second block of terms (lines 10 to 15) aims to identify studies with a focus on patients. Line 16 combines the MeSH terms for the *patient* concept with the Boolean operator "OR". Line 17 combines the above two concepts with the Boolean operator "AND".

Lines 17 to 20 locate studies that encompass both the *decision support* and *patient* concepts. Line 21 combines lines 17 to 20 with the Boolean operator "OR" to fully capture patient-specific decision support.

The last block of MeSH and free text terms (lines 22 to 30) identifies computer-based studies. Line 31 combines the MeSH and free text terms for the *computer* concept with the Boolean operator "OR".

Line 32 combines all three concepts with the Boolean operator "AND". Line 33 limits the search to studies published in the English language.

The search strategy has been translated for use in other databases (specifically, Embase, CINAHL and CENTRAL) by choosing relevant controlled vocabulary and free text terms.

Embase

Database: Embase <1996 to 2013 Week 41>
Search Strategy:
----
1 decision support system/ (11412)
2 decision making/ (108674)
3 medical information/ (47367)
4 audiovisual aid/ (102)
5 decision support*.tw. (7691)
6 decision tool*.tw. (462)
7 1 or 2 or 3 or 4 or 5 or 6 (167891)
8 patient education/ (67770)
9 consumer/ (24768)
10 patient participation/ (13738)
11 patient satisfaction/ (77558)
12 patient preference/ (3962)
13 8 or 9 or 10 or 11 or 12 (179777)
14 7 and 13 (11094)
15 patient decision making/ (4481)
16 decision aid*.tw. (1499)
17 shared decision making.tw. (2432)
18 patient decision*.tw. (1017)

19 14 or 15 or 16 or 17 or 18 (18566)

20 computer interface/ (20638)

21 medical informatics/ (12670)

22 internet/ (72563)

23 computer/ (23206)

24 microcomputer/ (6090)

25 interactive.tw. (29648)

26 internet.tw. (35108)

27 web based.tw. (17705)

28 computer*.tw. (170481)

29 20 or 21 or 22 or 23 or 24 or 25 or 26 or 27 or 28 (308559)

30 19 and 29 (2176)

31 limit 30 to English language (2083)

CINAHL

Query: Limiters/Expanders (Results)

S1 (MH "Decision Support Techniques+"): Search modes - Boolean/Phrase (3,261)

S2 (MH "Decision Support Systems, Clinical"): Search modes - Boolean/Phrase (2,304)

S3 (MH "Decision Making"): Search modes - Boolean/Phrase (25,143)

S4 (MH "Consumer Health Information"): Search modes - Boolean/Phrase (8,587)

S5 "decision support*": Search modes - Boolean/Phrase (5,944)

S6 "decision tool*": Search modes - Boolean/Phrase (108)

S7 S1 OR S2 OR S3 OR S4 OR S5 OR S6: Search modes - Boolean/Phrase (40,012)

S8 (MH "Patient Education+"): Search modes - Boolean/Phrase (53,689)

S9 (MH "Health Education+"): Search modes - Boolean/Phrase (85,801)

S10 (MH "Consumer Participation"): Search modes - Boolean/Phrase (11,581)

S11 (MH "Patient Satisfaction"): Search modes - Boolean/Phrase (30,664)

S12 S8 OR S9 OR S10 OR S11: Search modes - Boolean/Phrase (124,116)

S13 S7 AND S12: Search modes - Boolean/Phrase (4,121)

S14 (MH "Decision Making, Patient"): Search modes - Boolean/Phrase (10,071)

S15 "decision aid*": Search modes - Boolean/Phrase (608)

S16 "shared decision making": Search modes - Boolean/Phrase (1,077)

S17 "patient decision*": Search modes - Boolean/Phrase (352)

S18 S13 OR S14 OR S15 OR S16 OR S17: Search modes - Boolean/Phrase (15,036)

S19 (MH "User-Computer Interface+"): Search modes - Boolean/Phrase (6,946)

S20 (MH "Medical Informatics"): Search modes - Boolean/Phrase (2,327)

S21 (MH "Internet"): Search modes - Boolean/Phrase (30,199)

S22 (MH "Computers and Computerization"): Search modes - Boolean/Phrase (8,062)

S23 (MH "Computers, Portable+"): Search modes - Boolean/Phrase (3,365)

S24 "interactive": Search modes - Boolean/Phrase (12,463)

S25 "internet": Search modes - Boolean/Phrase (37,096)

S26 "web based": Search modes - Boolean/Phrase (5,185)

S27 "computer*": Search modes - Boolean/Phrase (98,570)

S28 S19 OR S20 OR S21 OR S22 OR S23 OR S24 OR S25 OR S26 OR S27: Search modes - Boolean/Phrase (143,311)
S29 S18 AND S28: Search modes - Boolean/Phrase (1,118)
S30 S29: Limiters - English Language (1,107)

CENTRAL

Search Name: CENTRAL PDA
Date Run: 16/10/13 19:20:08.795
Description: limited to trials

ID Search (Hits)
#1 MeSH descriptor: [Decision Support Techniques] this term only (1399)
#2 MeSH descriptor: [Decision Support Systems, Clinical] this term only (259)
#3 MeSH descriptor: [Decision Making] this term only (1415)
#4 MeSH descriptor: [Choice Behavior] this term only (717)
#5 MeSH descriptor: [Health Communication] this term only (23)
#6 MeSH descriptor: [Audiovisual Aids] this term only (242)
#7 decision support:ti,ab,kw (Word variations have been searched) (2849)
#8 decision tool:ti,ab,kw (Word variations have been searched) (460)
#9 #1 or #2 or #3 or #4 or #5 or #6 or #7 or #8 (5105)
#10 MeSH descriptor: [Patient Education as Topic] this term only (5917)
#11 MeSH descriptor: [Patients] explode all trees (3556)
#12 MeSH descriptor: [Consumer Participation] this term only (187)
#13 MeSH descriptor: [Patient Participation] this term only (776)
#14 MeSH descriptor: [Patient Satisfaction] this term only (7718)
#15 MeSH descriptor: [Patient Preference] this term only (212)
#16 #10 or #11 or #12 or #13 or #14 or #15 (17032)
#17 #9 and #16 (664)
#18 decision aid:ti,ab,kw (Word variations have been searched) (580)
#19 shared decision making:ti,ab,kw (Word variations have been searched) (250)
#20 patient decision:ti,ab,kw (Word variations have been searched) (4997)
#21 #17 or #18 or #19 or #20 (5386)
#22 MeSH descriptor: [User-Computer Interface] this term only (795)
#23 MeSH descriptor: [Medical Informatics] this term only (45)
#24 MeSH descriptor: [Internet] this term only (1448)
#25 MeSH descriptor: [Computers] this term only (427)
#26 MeSH descriptor: [Computers, Handheld] this term only (115)
#27 interactive:ti,ab,kw (Word variations have been searched) (1767)
#28 internet:ti,ab,kw (Word variations have been searched) (2908)
#29 web based:ti,ab,kw (Word variations have been searched) (1312)
#30 computer:ti,ab,kw (Word variations have been searched) (13527)
#31 #22 or #23 or #24 or #25 or #26 or #27 or #28 or #29 or #30 (17406)
#32 #21 and #31 (691)
#33 #32 in Trials (546)
