# Supplementary material for: Features of Computer-Based Decision Aids: Systematic Review, Thematic Synthesis, and Meta-Analyses
Source: J Med Internet Res. 2016 Jan 26;18(1):e20. doi: 10.2196/jmir.4982 (PMC4748141; doi:10.2196/jmir.4982)
Supplement: Multimedia Appendix 2 [file jmir_v18i1e20_app2.pdf]

Multimedia Appendix 2. Information about studies included in the systematic review

**Author, Year (Country)** Development manuscript / link to decision aid	**Study design**	**Decision context**	**Interventions** (Sample size)	**Control** (Sample size)	**Features being tested** (Types of components)†	**Quality of decision-making outcome** (SMDs provided for RCTs reporting knowledge or decisional conflict)
**TREATMENT**						
**Akl, 2007 [32]** (United States)	Pre/post	Chronic obstructive pulmonary disease treatment	Computer-based decision aid (N=8)	Baseline measures	CC(abcd), T(c), PN(b), EVC(c), F(d)	Knowledge<br>Decisional conflict
**Berman, 2011 [33]** (United States)	Pre/post	Abdominal aortic aneurysm treatment	Computer-based decision aid (N=12)	Baseline measures	CC(bc), T(b), PN(a), F(b), SS(bc)	Knowledge<br>Decisional conflict
***Berry, 2013 [34]** (United States)<br>*Berry, 2010* **[35]**	RCT	Prostate cancer treatment	Web-based decision aid + usual patient education + web links (n=218)	Usual patient education + web links (n=182)	CC(a), T(abc), PN(b), F(f), SS(c)	Decisional conflict (SMD=0.08)<br>Decisional certainty
**Brink, 2000[a] [36]** (United States)	Pre/post	Prostate cancer treatment	CD-ROM decision aid (N=43)	Baseline measures	CC(ab), T(b), PN(a), EVC(ac), F(d), SS(b)	Knowledge<br>Decisional self-efficacy
***Davison, 2007 [31]** (Canada)	RCT	Prostate cancer treatment	Tailored risk summary + videotape + written information package (n=148)	Videotape + written information package (n=145)	T(bc), F(f)	Decisional conflict (SMD=0.04)<br>Satisfaction with decision making
***de Achaval, 2012 [37]** (United States)	RCT	Knee osteoarthritis treatment	1: Videobooklet + values clarification exercise (n=69)<br>2: Videobooklet (n=70)	Educational booklet (n=69)	1: PN(a), EVC(cd), F(cf)<br>2: PN(a)	Decisional conflict (SMD=0.37)
**Deyo, 2000[b] [38]** (United States)<br>*Spunt, 1996* **[39]**<br>*Phelan, 2001* **[40]**	RCT	Back pain treatment	Videodisc + booklet (n=190)	Booklet (n=203)	CC(abc), T(ab), PN(a), F(f), SS(c)	Satisfaction with decision making
***Diefenbach, 2012 [30]** (United States)<br>Diefenbach, 2004 **[41]**	RCT	Prostate cancer treatment	Computer-based decision aid (n=53)	Standard brochures (n=19)	CC(ab), T(ab[c][+]), PN(a), EVC(b, unclear), F(af), SS(c)<br>[+]sometimes included	Decisional conflict (SMD=0.62)
***Feldman-Stewart, 2012 [42]** (Canada)<br>*Feldman-Stewart, 2006* **[43]**	RCT	Prostate cancer treatment	Computer-based decision aid with values clarification (n=69)	Computer-based decision aid (n=65)	T(c), EVC(c), F(d)	Decisional conflict (SMD=0.10)
**Fraenkel, 2007 [44]** (United States)	RCT	Knee pain treatment	Computer-based decision aid (n=44)	Information pamphlet (n=40)	EVC(d), F(cdf)	Decisional self-efficacy
**Fraenkel, 2012 [45]** (United States)	Pre/post	Rheumatoid arthritis treatment	Web-based decision aid (N=104)	Baseline measures	CC(abc), EVC(c), F(bd)	Knowledge<br>Decisional conflict
**Heller, 2008 [46]** (United States)	RCT	Breast cancer treatment	CD-ROM decision aid (n=67)	Usual care (n=66)	CC(a), T(c), PN(a), SS(c)	Knowledge (Insufficient data to calculate SMD)<br>Satisfaction with decision making

**Jibaja-Weiss, 2006[c] [47]**<br>(United States)<br>*Development manuscript not accessible*	Pre/post	Breast cancer treatment	Computer-based decision aid (N=44)	Baseline measures	CC(abc), T(abc), PN(ab), EVC(bc), F(f), SS(abc)	Decisional conflict
***Jibaja-Weiss, 2011[c] [48]**<br>(United States)<br>*Jibaja-Weiss, 2006* **[47]**<br>*Link was updated; not used in abstraction*	RCT	Breast cancer treatment	Computer-based decision aid (n=44)	Usual care (n=39)	CC(abc), T(abc), PN(ab), EVC(bc), F(f), SS(abc)	Knowledge (SMD=0.75)<br>Decisional conflict (SMD=0.25)<br>Satisfaction with decision making<br>Decisional certainty
**Liao, 1996[d] [49]**<br>(United States)	Pre/post	Ischemic heart disease treatment	Videodisc decision aid (N=60)	Baseline measures	CC(ab), T(b), PN(a)	Decisional certainty
**Maslin, 1998 [50]**<br>(United Kingdom)	RCT	Breast cancer treatment	Videodisc (n=51)	Usual care (n=49)	CC(c), T(ab), SS(c)	Satisfaction with decision making
**Molenaar, 2001 [51]**<br>(Netherlands)	RCT	Breast cancer treatment	CD-ROM decision aid (n=87)	Usual care (n=72)	CC(abcd), PN(a), SS(ac)	Satisfaction with decision making
**Morgan, 2000[d] [52]**<br>(Canada)<br>*Liao, 1996* **[49]**	RCT	Ischemic heart disease treatment	Videodisc (n=90)	Usual care (n=97)	CC(ab), T(ab), PN(a), F(f), SS(c)	Knowledge (Insufficient data to calculate SMD)<br>Satisfaction with decision making
***Murray, 2001 [53]**<br>(United Kingdom)<br>*Clinician guide not accessible*	RCT	Menopausal symptom treatment	Videodisc decision aid + booklet (n=94)	Usual care (n=96)	CC(bc), SS(c)	Decisional conflict (SMD=0.54)<br>Decisional certainty
**Phelan, 2001[b] [40]**<br>(United States)<br>*Spunt, 1996* **[39]**	RCT	Back pain treatment	Videodisc decision aid + booklet (n=41)	Booklet (n=49)	CC(abc), T(ab), PN(a), F(f)	Knowledge (Insufficient data to calculate SMD)
**Piercy, 1999 [54]**<br>(Canada)<br>*Development manuscript not accessible*	Pre/post	Benign prostatic hyperplasia treatment	Videodisc decision aid (N=635)	Baseline measures	CC(c), T(ab), PN(a), F(f), SS(c)	Decisional certainty
***Protheroe, 2007 [55]**<br>(United Kingdom)	RCT	Menorrhagia treatment	Computerized decision aid + written information (n=54 for knowledge; n=69 for decisional conflict)	Written information (n=54 for knowledge; n=69 for decisional conflict)	CC(bd), EVC(bcd), F(df)	Knowledge (SMD=0.57)<br>Decisional conflict (SMD=1.04)<br>Decisional certainty
***Rostom, 2002 [56]**<br>(Canada)	RCT	Menopausal symptom treatment	Computer-based decision aid (n=25)	Audiobooklet decision aid (n=26)	CC(b), T(unclear), F(b)	Knowledge (SMD=0.64)
**Sawka, 2011 [57]**<br>(Canada)<br>*Sawka, 2011* **[58]**	Pre-post	Papillary thyroid cancer treatment	Web-based decision aid (N=50)	Baseline measures	CC(bcd), SS(a)	Knowledge
***Sawka, 2012 [59]**<br>(Canada)<br>*Sawka, 2011* **[58]**	RCT	Papillary thyroid cancer treatment	Web-based decision aid (n=37)	Usual care (n=37)	CC(bcd), SS(a)	Knowledge (SMD=1.88)<br>Decisional conflict (SMD=1.48)<br>Decisional certainty
***Schapira, 2007 [60]**<br>(United States)	RCT	Menopausal symptom treatment	Computer-based decision aid (n=85)	Printed pamphlet (n=86)	CC(ad), T(ab), PN(a)	Knowledge (SMD=-0.15)<br>Decisional conflict (SMD=0.07)<br>Satisfaction with decision making
***Simon, 2012 [61]**<br>(Germany)	RCT	Depression or back pain treatment	Web-based decision aid (n=308)	Static patient information (n=349)	CC(bc), T(abc), SS(c)	Knowledge (SMD=0.23)<br>Decisional conflict (SMD=0.24)

**Sivell, 2012 [62]**<br>(United Kingdom)<br>*Sivell, 2012* **[63]**<br>*Webpage data abstracted; however, link not currently accessible (update in progress)*	Pre/post	Breast cancer treatment	Web-based decision aid (n=45)	Baseline measures (n=54)	CC(abcd), PN(ab), EVC(c), F(df), SS(ac)	Knowledge<br>Decisional certainty
**Spunt, 1996[b] [39]**<br>(United States)	Pre/post	Back pain treatment	Videodisc decision aid (N=175)	Baseline measures	CC(abc), T(ab), PN(a), F(f)	Decisional certainty
**Taylor, 2010[a] [64]**<br>(United States)	RCT	Prostate cancer treatment	CD-ROM decision aid + decision tools (n=42)	CD-ROM decision aid (n=53)	CC(b), T(bc), EVC(b)	Satisfaction with decision making
***Thomson, 2007 [65]**<br>(United Kingdom)<br>*Thomson, 2002* **[66]**	RCT	Atrial fibrillation treatment	Computer-based decision aid (n=53)	Paper guidelines (n=55)	T(abc), EVC(c), F(def), SS(c)	Knowledge (SMD=0.06)<br>Decisional conflict (SMD=0.42)
**van Til, 2010 [67]**<br>(Netherlands)	Pre/post	Ankle-foot impairment treatment after stroke or arm-hand function treatment after cervical spinal cord injury	Web-based decision aid (n=39 for stroke; n=38 for spinal injury)	Baseline measures	EVC(unclear)	Knowledge<br>Decisional conflict
**RISK MANAGEMENT**						
**Hooker, 2011[e] [68]**<br>(United States)<br>*Kaufman, 2003* **[69]**	RCT	Breast cancer risk management	CD-ROM decision aid (n=96)	Usual care (n=108)	CC(abc), T(abc), PN(a), EVC(cd), F(de), SS(c)	Decisional certainty
***Huyghe, 2009 [70]**<br>(France)	RCT	Fertility risk management	Computer-based decision aid (n=10)	Usual care (n=10)	CC(abd), EVC(c), F(b), SS(bc)	Knowledge (SMD=0.51)<br>Decisional conflict (SMD=1.23)
***Montgomery, 2007 [71]**<br>(United Kingdom)<br>*Emmett, 2007* **[72]**	RCT	Vaginal birth after cesarean risk management	1: Computer-based decision aid (n=198 for knowledge and decisional conflict)<br>2: Computer-based information program (n=196 for knowledge; n=201 for decisional conflict)	Usual care (n=202 for knowledge; n=201 for decisional conflict)	1: CC(ab), EVC(c), F(df)<br>2: CC(abd)	Knowledge (SMD=0.57)<br>Decisional conflict (SMD=0.28)<br>Satisfaction with decision making
***Saver, 2007 [73]**<br>(United States)	RCT	Menopausal risk management	*RCT 1*: Web-based decision aid (n=144)<br>*RCT 2*: Web-based decision aid (n=22)	*RCT 1*: Brochure (n=199)<br>*RCT 2*: Usual care (n=19)	CC(bd), EVC(b), SS(a)	Knowledge (SMD=0.49)<br>Decisional conflict (SMD=-0.02)<br>Satisfaction with decision making
**Schwartz, 2009[e] [74]**<br>(United States)<br>*Kaufman, 2003* **[69]**	RCT	Breast cancer risk management	CD-ROM decision aid (n=110)	Usual care (n=114)	CC(abc), T(abc), PN(a), EVC(cd), F(de), SS(c)	Decisional conflict (Insufficient data to calculate SMD)<br>Satisfaction with decision making<br>Decisional certainty
***Wilkie, 2013 [75]**<br>(United States)<br>*Gallo, 2013* **[76]**	RCT	Reproductive health risk management in people with sickle cell disease or trait	Web-based decision aid (n=114)	Web-based e-Book (n=117)	CC(b), T(ab), PN(ab), EVC(ef), SS(b)	Knowledge (SMD=0.43)

**SCREENING**						
**Allen, 2009 [77]**<br>(United States)	Pre/post	Prostate cancer screening	Computer-based decision aid (N=107)	Baseline measures	CC(abc), EVC(unclear), F(f)	Knowledge<br>Decisional conflict<br>Decisional certainty<br>Decisional self-efficacy
***Allen, 2010 [78]**<br>(United States)<br>*Development manuscript not accessible*	RCT	Prostate cancer screening	Computer-based decision aid (n=291)	Usual care (n=334)	CC(abc), T(ab), PN(b), EVC(c), F(ef)	Knowledge (SMD=0.19)<br>Decisional conflict (SMD=0.17)<br>Decisional certainty<br>Decisional self-efficacy
***Ellison, 2008 [79]**<br>(United States)<br>*Link no longer accessible*	RCT	Prostate cancer screening	Enhanced web-based decision aid (n=46)	Standard web-based decision aid (n=41)	CC(b), T(a), PN(a), SS(a)	Knowledge (SMD=0.58)
**Evans, 2010 [80]**<br>(United Kingdom)<br>*Link no longer accessible*	RCT	Prostate cancer screening	1: Web-based decision aid (n=48)<br>2: Paper-based decision aid (n=57)	1: Usual care (n=69)<br>2: Usual care at 6 months (n=100)	1: PN(a), EVC(c)<br>2: no multimedia features	Knowledge (Insufficient data to calculate SMD)<br>Decisional conflict (Insufficient data to calculate SMD)
***Frosch, 2008 [81]**<br>(United States)	RCT	Prostate cancer screening	1: Traditional decision aid + chronic disease trajectory model (n=152)<br>2: Chronic disease trajectory model (n=153)<br>3: Traditional decision aid (n=155)	Internet links (n=151)	1: PN(a), EVC(cd), SS(c)<br>2: EVC(cd)<br>3: PN(a), SS(c)	Knowledge (SMD=0.25)<br>Decisional conflict (SMD=0.07)
**Green, 2004 [82]**<br>(United States)<br>*Development manuscript not accessible*	RCT	BRCA1/2 screening	CD-ROM decision aid (n=56)	Usual care (n=61)	CC(a)	Knowledge (Insufficient data to calculate SMD)<br>Satisfaction with decision making
**Kuppermann, 2009 [83]**<br>(United States)	RCT	Prenatal screening	Computer-based decision aid (n=244)	Online educational booklet (n=252)	T(c, unclear), EVC(unclear), F(f)	Knowledge (Insufficient data to calculate SMD)<br>Decisional conflict (Insufficient data to calculate SMD)
**Leung, 2004 [84]**<br>(Hong Kong)	RCT	Prenatal screening	Computerized decision aid + video + information leaflet (n=99)	Video + information leaflet (n=101)	CC(bc), T(c)	Decisional certainty
**Lindblom, 2012 [85]**<br>(Australia)<br>*Link no longer accessible*	Pre/post	Colorectal cancer screening	Web-based decision aid (N=81)	Baseline measures	T(c), F(b)	Knowledge
***Manne, 2010 [86]**<br>(United States)	RCT	Lynch syndrome screening	CD-ROM decision aid + education session (n=92)	Education session only (n=95)	CC(bc)	Knowledge (SMD=1.09)<br>Decisional conflict (SMD=0.36)<br>Satisfaction with decision making<br>Decisional self-efficacy
***Mathieu, 2010 [87]**<br>(Australia)<br>*www.mammogram.med.usyd.edu.au* **[88]**	RCT	Breast cancer screening	Computer-based decision aid (n=113)	Usual care (n=189)	CC(acd), PN(a), EVC(c), F(f)	Knowledge (SMD=0.59)<br>Decisional certainty

**Ruffin, 2007 [89]**<br>(United States)<br>*Link no longer accessible*	RCT	Colorectal cancer screening	Computer-based decision aid (n=87)	Standard website (n=87)	EVC(d), F(d), SS(c)	Decisional certainty
**Rupert, 2013 [90]**<br>(United States)	Pre/post	Hereditary breast and ovarian cancer syndrome screening	Web-based decision aid (N=48)	Baseline measures	CC(abd), T(ab), EVC(a), F(df), SS(c)	Knowledge<br>Decisional self-efficacy
***Schroy, 2011 [91]**<br>(United States)<br>*www.yourdiseaserisk.wustl.edu* **[92]**	RCT	Colorectal cancer screening	1: Computer-based decision aid + personalized risk assessment (n=223)<br>2: Computer-based decision aid (n=212)	Webpage (n=231)	1: CC(abc), T(ab), PN(a), EVC(d), SS(c)<br>2: CC(abc), PN(a), EVC(d), SS(c)	Knowledge (SMD=0.91)<br>Satisfaction with decision making
***Volk, 2008 [93]**<br>(United States)	RCT	Prostate cancer screening	Computer-based decision aid (n=38 for low literacy; n=108 for high literacy)	Audio-booklet (low literacy n=46; high literacy n=131)	CC(ab), T(a), PN(b), EVC(e), SS(a)	Knowledge (Insufficient data to calculate SMD)<br>Decisional conflict (SMD=0.33)
**PREVENTION**						
***Banegas, 2013 [94]**<br>(United States)<br>*Fagerlin, 2011* **[95]**	RCT	Breast cancer prevention	Web-based decision aid (n=690)	Usual care (n=160)	T(ab), EVC(unclear)	Decisional conflict (SMD=0.56)<br>Decisional certainty
***Ozanne, 2007 [96]**<br>(United States)	RCT	Breast cancer prevention	Computer-based decision aid (n=15)	Usual care (n=15)	T(ab)	Knowledge (SMD=0.63)<br>Decisional conflict (SMD=0.78)<br>Satisfaction with decision making<br>Decisional certainty
**Sheridan, 2006[f] [97]**<br>(United States)<br>*Pignone, 2004* **[98]**<br>*Link was updated; not used in abstraction*	RCT	Coronary heart disease prevention	Web-based decision aid (n=41)	Risk factor list (n=34)	T(abc), F(f), SS(c)	Decisional certainty
**Sheridan, 2010[f] [99]**<br>(United States)<br>*Pignone, 2004* **[98]**<br>*www.med-decisions.com/h2hv2* **[100]**	RCT	Coronary heart disease prevention	Web-based decision aid + values clarification (n=75)	Web-based decision aid (n=62)	EVC(cd), F(c)	Knowledge (Insufficient data to calculate SMD)<br>Decisional certainty<br>Decisional self-efficacy
**Sheridan, 2011[f] [101]**<br>(United States)<br>*Pignone, 2004* **[98]**<br>*Sheridan, 2010* **[99]**<br>*www.med-decisions.com/h2hv2* **[100]**	RCT	Coronary heart disease prevention	Web-based decision aid (n=77)	Risk factor list (n=77)	CC(cd), T(abc), EVC(cd), F(cf), SS(c)	Decisional certainty

*Studies included in the meta-analysis; [a-f] Studies employing the same decision aids are denoted with alpha superscripts (a through f); it is important to note, however, that the studies may be testing different features of the decision aid (as determined by the control group) or updated versions of the same decision aid; †Legend for column: CC = content control (with types: a = navigation, b = clarity of information, c = optional information, d = access to external resources), T = tailoring (with types: a = demographics, b = clinical condition, c = values, preferences, and beliefs, d = knowledge deficits), PN = patient narratives (with types: a = patient stories, b = behavior modeling), EVC = explicit values clarification (with types: a = decision points, b = notebook, c = weighting exercises, d = trade-off exercises, e = social matching, f = personal reflection), F = feedback (with types: a = decision aid progress, b = knowledge, c = summary of preferences, d = optimal choice, e = decisional consistency, f = summary of decision aid activity), and SS = social support (with types: a = community support, b = integration of family, c = facilitation of shared decision making).
